# Increasing carbohydrate oxidation improves contractile reserves and prevents hypertrophy in porcine right heart failure

**DOI:** 10.1038/s41598-020-65098-7

**Published:** 2020-05-18

**Authors:** Nikolaj Bøgh, Esben S. S. Hansen, Camilla Omann, Jakob Lindhardt, Per M. Nielsen, Robert S. Stephenson, Christoffer Laustsen, Vibeke E. Hjortdal, Peter Agger

**Affiliations:** 1 The Department of Cardiothoracic and Vascular Surgery, Aarhus University Hospital, Palle Juul-Jensens Boulevard 99, 8200 Aarhus N, Denmark; 2 The MR Research Centre, Department of Clinical Medicine, Aarhus University, Palle Juul-Jensens Boulevard 99, 8200 Aarhus N, Denmark; 3 Comparative Medicine Lab, Department of Clinical Medicine, Aarhus University, Palle Juul-Jensens Boulevard 99, 8200 Aarhus N, Denmark; 4 Institute of Clinical Sciences, College of Medical and Dental Science, The University of Birmingham, Birmingham, United Kingdom

**Keywords:** Cardiac hypertrophy, Heart failure

## Abstract

In heart failure, myocardial overload causes vast metabolic changes that impair cardiac energy production and contribute to deterioration of contractile function. However, metabolic therapy is not used in heart failure care. We aimed to investigate the interplay between cardiac function and myocardial carbohydrate metabolism in a large animal heart failure model. Using magnetic resonance spectroscopy with hyperpolarized pyruvate and magnetic resonance imaging at rest and during pharmacological stress, we investigated the *in-vivo* cardiac pyruvate metabolism and contractility in a porcine model of chronic pulmonary insufficiency causing right ventricular volume overload. To assess if increasing the carbohydrate metabolic reserve improves the contractile reserve, a group of animals were fed dichloroacetate, an activator of pyruvate oxidation. Volume overload caused heart failure with decreased pyruvate dehydrogenase flux and poor ejection fraction reserve. The animals treated with dichloroacetate had a larger contractile response to dobutamine stress than non-treated animals. Further, dichloroacetate prevented myocardial hypertrophy. The *in-vivo* metabolic data were validated by mitochondrial respirometry, enzyme activity assays and gene expression analyses. Our results show that pyruvate dehydrogenase kinase inhibition improves the contractile reserve and decreases hypertrophy by augmenting carbohydrate metabolism in porcine heart failure. The approach is promising for metabolic heart failure therapy.

## Introduction

**Figure 1:**
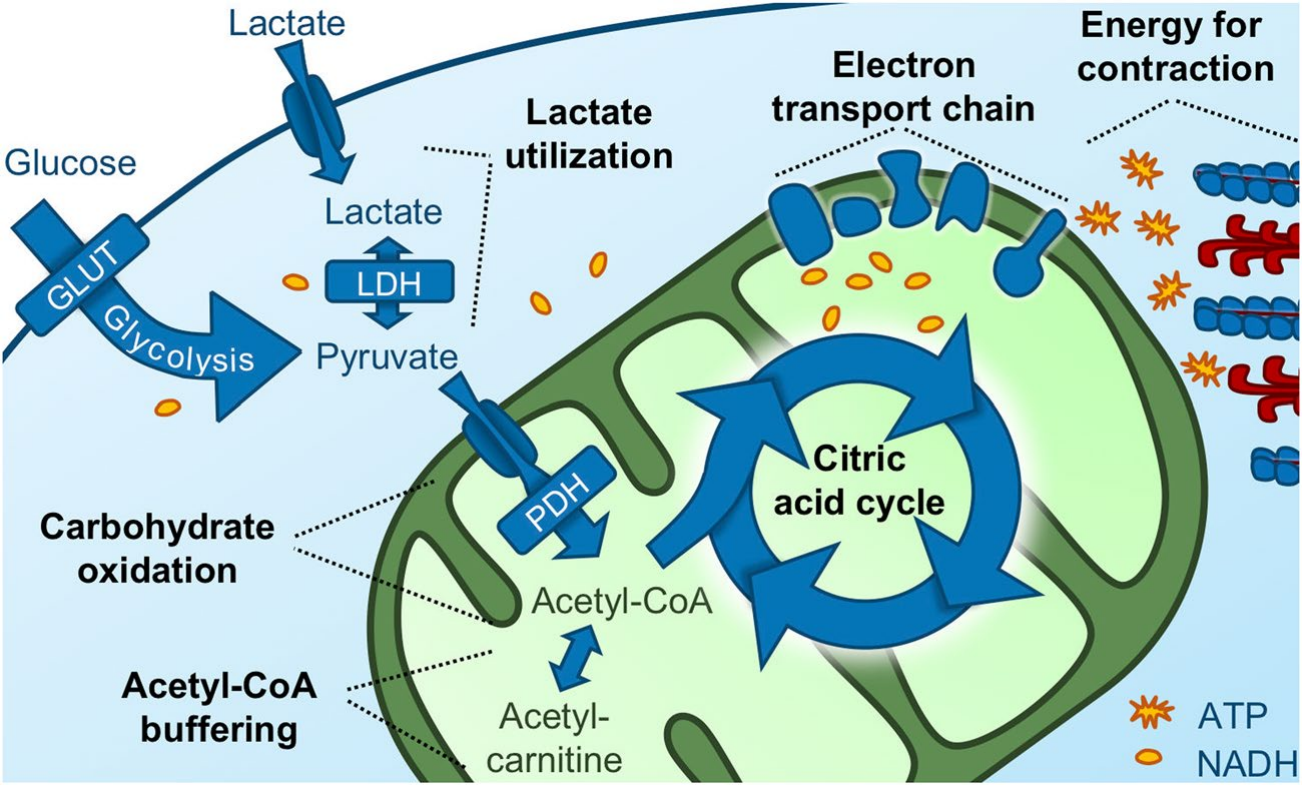
Overview of the cytosolic and mitochondrial carbohydrate metabolic pathways involved in the progression of heart failure. The illustrated pathways are the major carbohydrate sources of the energy that is ultimately needed for myocardial contraction, and they thus represent potential targets for future metabolic therapy. Fatty-acid metabolism is omitted. GLUT = glucose transporters, LDH = lactate dehydrogenase, PDH = pyruvate dehydrogenase.

Clinically undetectable metabolic changes contribute to the cardiac dysfunction in heart failure^1,2^. Flux through the pyruvate dehydrogenase (PDH) is decreased despite increased rates of glycolysis. This is often referred to as uncoupling of glycolysis and carbohydrate oxidation^2–5^. PDH flux is regulated by several mechanisms, including the pyruvate dehydrogenase kinases (PDKs) and phosphatases. In addition, utilization of fatty acids inhibits carbohydrate oxidation through the Randle cycle^1^. Recoupling glycolysis to carbohydrate oxidation by any means improves function^6–8^. Further downstream, the citric acid cycle, the electron transport chain and the phosphorous apparatus, three essential parts of mitochondrial metabolism, are also impaired in heart failure^1,9^. Thus, both mitochondrial and cytosolic carbohydrate metabolic pathways are affected in late-stage disease (Fig. 1). Metabolism has been suggested a pharmacological target for decades^10^. However, as studies of early-stage heart failure metabolism are few, we do not understand which changes are directly involved in disease progression and which are non-causal bystanders. This has hindered development of metabolic therapies^1,2^.

Impaired myocardial metabolism limits contraction, particularly during the increased energy demands of exercise. Poor metabolic reserves, defined as small capacity to increase energy production upon demand, are associated with poor contractile reserves^1,2,9,11^. A small contractile reserve, defined as a small ejection fraction increase from rest to exercise or pharmacological stress, is associated with poor prognosis and likely reduces exercise capacity^12,13^. Increasing the metabolic reserve may improve exercise tolerance and the prognosis in heart failure. However, studies assessing the myocardial metabolic reserve and how it can be targeted to improve function are lacking.

The recent advent of hyperpolarized magnetic resonance techniques has enabled *in-vivo* metabolic assessment of the heart. Magnetic resonance spectroscopy (MRS) after infusion of hyperpolarized [1-^13^C]pyruvate allows for quantification of flux through the lactate dehydrogenase (LDH), the PDH and the alanine transaminase (ALT). Combined with conventional magnetic resonance imaging (MRI), MRS with hyperpolarized [1-^13^C]pyruvate allows quantification of metabolism, function and perfusion in one experimental setting.

Dichloroacetate (DCA) is a small molecule that inhibits PDK activity and thereby increases PDH flux. We hypothesized that recoupling glycolysis to mitochondrial metabolism with DCA increases the carbohydrate metabolic reserve and preserves the contractile reserve in experimental chronic heart failure. We investigated this matter in a porcine model of right ventricular volume overload (RVO) with MRI and hyperpolarized [1-^13^C]pyruvate MRS at rest and under pharmacological stress. Moreover, we supported the *in-vivo* investigations with *in-vitro* analyses of gene expression and mitochondrial function.

## Results

### DCA reduces hypertrophy and restores contractile reserve

**Figure 2:**
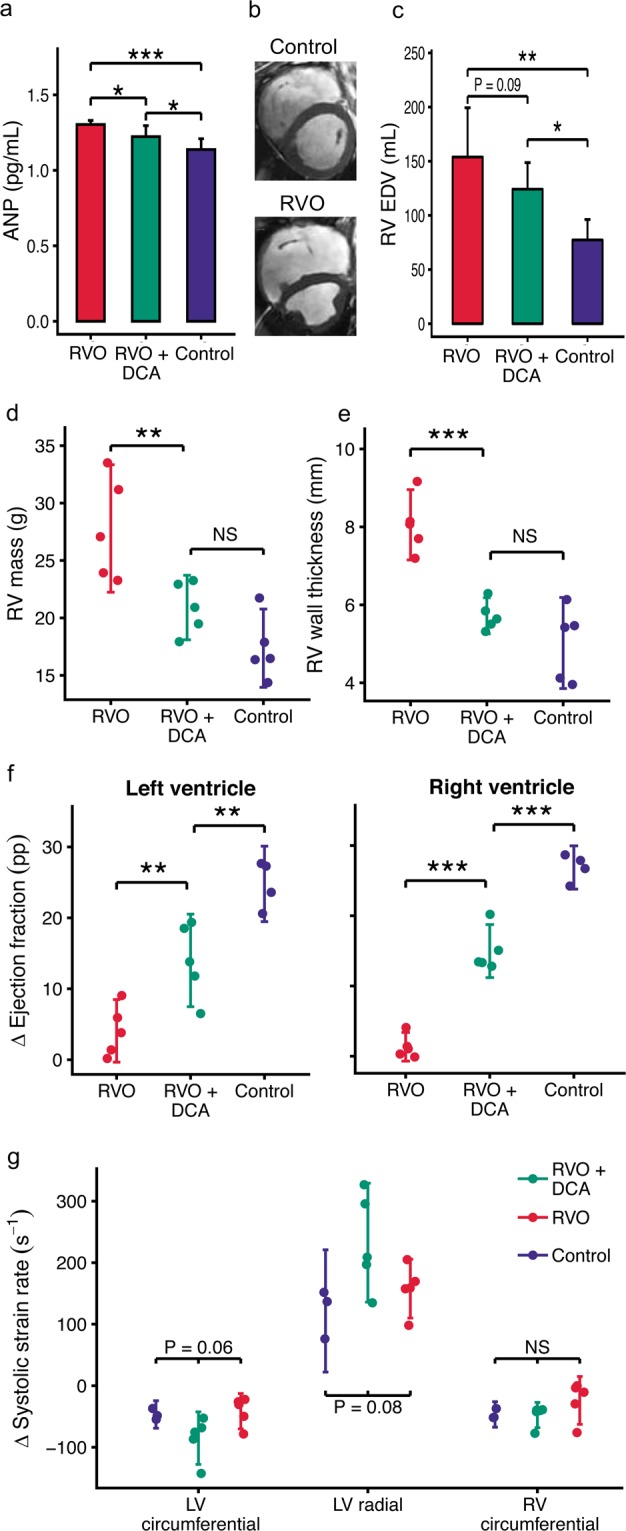
Dichloroacetate (DCA) attenuates hypertrophy and contractile impairment in pigs with right ventricular (RV) volume overload (RVO). (**a**): Atrial natriuretic peptide (ANP) in plasma. (**b + c**): CINE MRI shows dilation of the right ventricle after 19 weeks of overload, quantified as the end-diastolic volume (EDV). The white scale bars are 1 cm. (**d**) RV mass from MRI. (**e**) RV wall thickness *ex-vivo* after fixation. (**f + g**): MRI determined change in systolic function from rest to stress induced with dobutamine, pp indicates percentage points. Error bars indicate 95% confidence intervals. NS: P > 0.1, *P < 0.05, **P < 0.01, ***P < 0.001, (**g**) was tested with Kruskal-Wallis, (**a + c-f**) with ANOVA with Benjamini-Hochberg correction.

All animals survived without observable symptoms of heart failure. Both RVO groups had elevated plasma levels of atrial natriuretic peptide (ANP), however slightly reduced by DCA treatment (Fig. 2a). The DCA treated and the non-treated RVO groups had comparable pulmonary regurgitation fractions (~21%, Table S1). The RVO groups had larger right ventricular end-diastolic volume (Fig. 2b,c). The overload caused hypertrophy of the right ventricle (Fig. 2d,e). The non-treated RVO group had a right ventricular myocardial mass of 27.8 ± 4.5 g (P < 0.001 vs controls) while it was 20.9 ± 2.3 g in the DCA treated RVO group (P = 0.0095 vs RVO). The latter was not significantly different from the controls (17.4 ± 2.7 g). The non-treated group had hyperdynamic systolic function at rest, which was prevented in the DCA group (Tables 1 + S1). Under dobutamine stress (Fig. 2f,g, Table S2), the DCA treated group increased ejection fraction of both ventricles more than the non-treated RVO group (LV: 14 ± 5.2 vs 4.1 ± 3.6 percent points, P = 0.003; RV: 15 ± 3 vs 1.4 ± 1.6 percent points, P < 0.001).

**Table 1:** Rest and stress hemodynamics, whole-body metabolism and cardiac function in pigs subjected to chronic right ventricular volume overload (RVO) with and without dichloroacetate (DCA).

	Rest			Stress		
	RVO	RVO + DCA	Control	RVO	RVO + DCA	Control
	(n = 5)	(n = 5)	(n = 5)	(n = 5)	(n = 5)	(n = 4)
Vital parameters						
Heart rate	45.8 (9.8)	43.8 (6.5)	53.2 (4.8)	79.2 (7.9)	75.6 (4.3)	83 (9.4)
Mean arterial pressure (mmHg)	65 (3.1)	62.2 (5.9)	70.8 (6.6)	75.4 (7.1)	77 (9.9)	81.5 (4.7)
Venous plasma						
Glucose (mmol/L)	5.58 (2.7)	4.72 (0.5)	6.8 (1.6)	5.64 (2.6)	4.18 (0.9)	6.75 (1.9)
Lactate (mmol/L)	2.12^†^ (1.1)	0.64 (0.4)	1.54 (0.4)	2.42^†^ (0.8)	0.64 (0.4)	1.68 (0.7)
Free fatty acids (mmol/L)	0.46 (0.07)	0.48 (0.04)	0.51 (0.08)	0.99 (0.34)	1.15 (0.56)	1.08 (0.66)
Insulin (mIU/L)	1.26^*^ (0.62)	1.69^*^ (0.21)	0.48 (0.25)	1.42^*^ (0.7)	1.71^*^ (0.12)	0.48 (0.25)
MRI						
LV EF (%)	78.4^*†^ (5.5)	72.1^*^ (2)	60.4 (3.3)	82.4 (4.5)	86.1 (4.2)	84.7 (3.2)
RV EF (%)	71.3^*†^ (4.1)	63.3 (6)	58.7 (5)	72.7^*^ (4.6)	78.3^*^ (6.4)	87.2 (2.5)
Cardiac output (L/min)	3.4 (0.6)	2.9 (0.9)	2.6 (0.6)	6.2^*^ (1.2)	6.8^*^ (0.7)	9.4 (1.4)
LV perfusion (mL/100 mL/min)	330 (156)	270 (68.9)	322 (58.8)	299 (84)	338 (127)	332 (129)

Stress was induced with dobutamine (10 mcg/kg/min). Asterisks indicate significance vs control group and daggers indicate significance vs RVO + DCA group in ANOVA with Benjamini-Hochberg correction.

### DCA increases the pyruvate oxidation reserve

**Figure 3:**
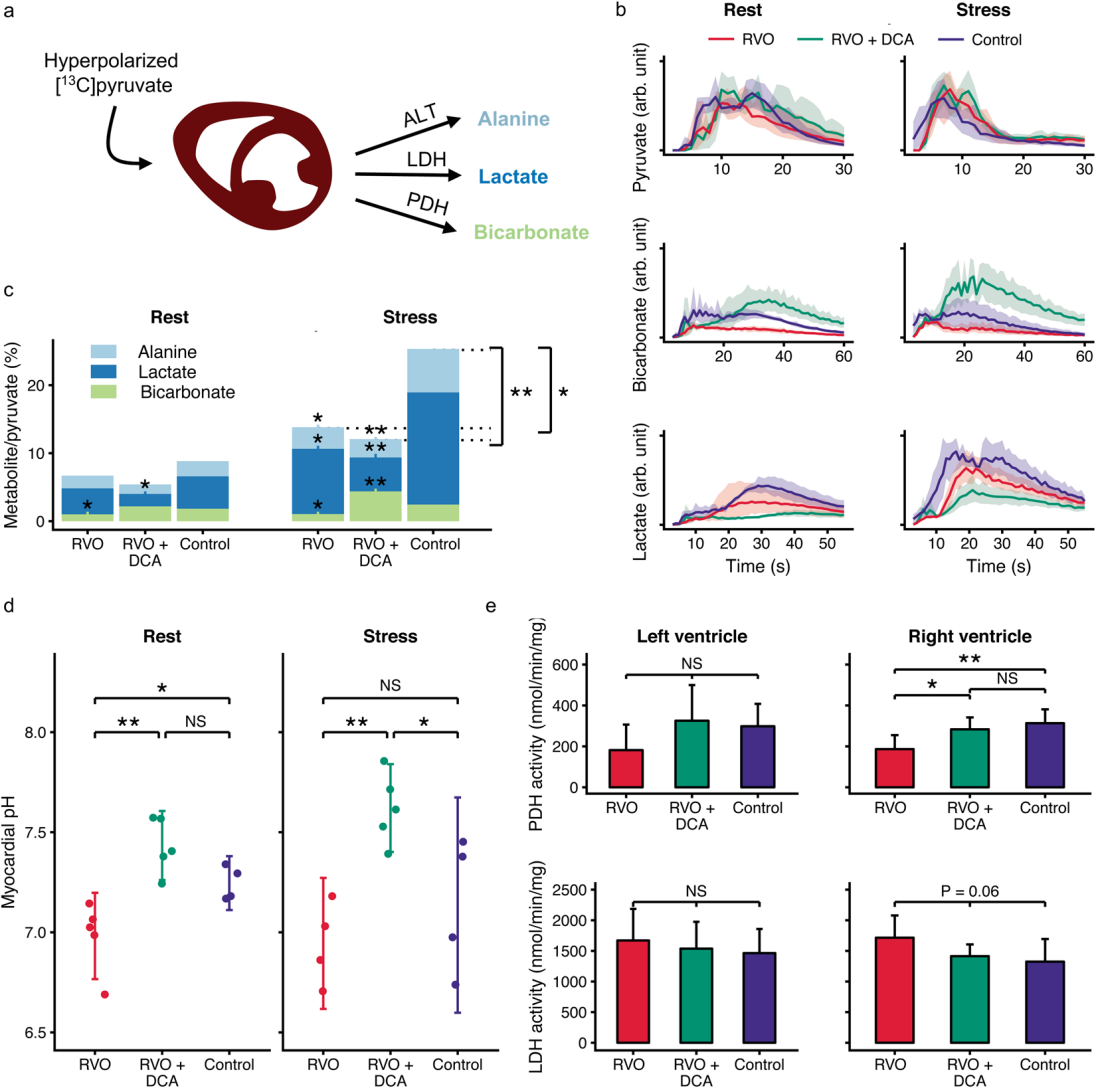
Dichloroacetate (DCA) reverts the deterioration of myocardial pyruvate metabolism after chronic right ventricular overload (RVO) in pigs. (**a**) We assessed metabolism with hyperpolarized [1-^13^C]pyruvate magnetic resonance spectroscopy under rest and dobutamine-induced stress. This technique allows assessment of flux through the pyruvate dehydrogenase (PDH), the lactate dehydrogenase (LDH) and the alanine transaminase (ALT). (**b**) The lactate and bicarbonate production of the heart after injection of hyperpolarized pyruvate. (**c**) The metabolism quantified using a model-free approach. The asterisks indicate significance vs. controls. Only significant comparisons are shown for display purposes. (**d**) Myocardial pH modelled from the spectroscopy data. e: *In-vitro* enzyme activity. Error bars and ribbons indicate 95% confidence intervals. NS: P > 0.1, *P < 0.05, **P < 0.01. ***P < 0.001, tested with ANOVA with Benjamini-Hochberg correction.

Using hyperpolarized [1-^13^C]pyruvate MRS (Fig. 3a + b), we observed a 45% decrease in resting PDH flux of the non-treated RVO group compared with controls (P = 0.044). This was normalised in the DCA treated RVO group (Fig. 3c). During stress, the non-treated RVO group showed a 56% decrease in PDH flux relative to controls (P = 0.024) while the DCA treated animals showed a 76% increase in PDH flux (P = 0.005). Despite similar myocardial perfusion (Table 1), the controls showed an increase in the sum of PDH, LDH and ALT flux during stress compared with the RVO groups (P < 0.05 vs both). At rest, the estimated myocardial pH (Fig. 3d) of the non-treated RVO group was significantly lower than in the DCA and control groups (P < 0.05 vs both). During stress, the DCA treated RVO group had a higher myocardial pH than the control and non-treated RVO groups (P < 0.05 vs both). *In-vitro* PDH activity (Fig. 3e) in the right ventricular myocardium was significantly increased in the DCA treated compared with the non-treated RVO group (P = 0.0147), while there was no difference between DCA treated animals and controls (P = 0.3807). A similar tendency was also observed in the left ventricle although this did not reach statistical significance (P = 0.19 vs non-treated RVO, P = 0.72 vs controls).

### DCA increases mitochondrial respiration

**Figure 4:**
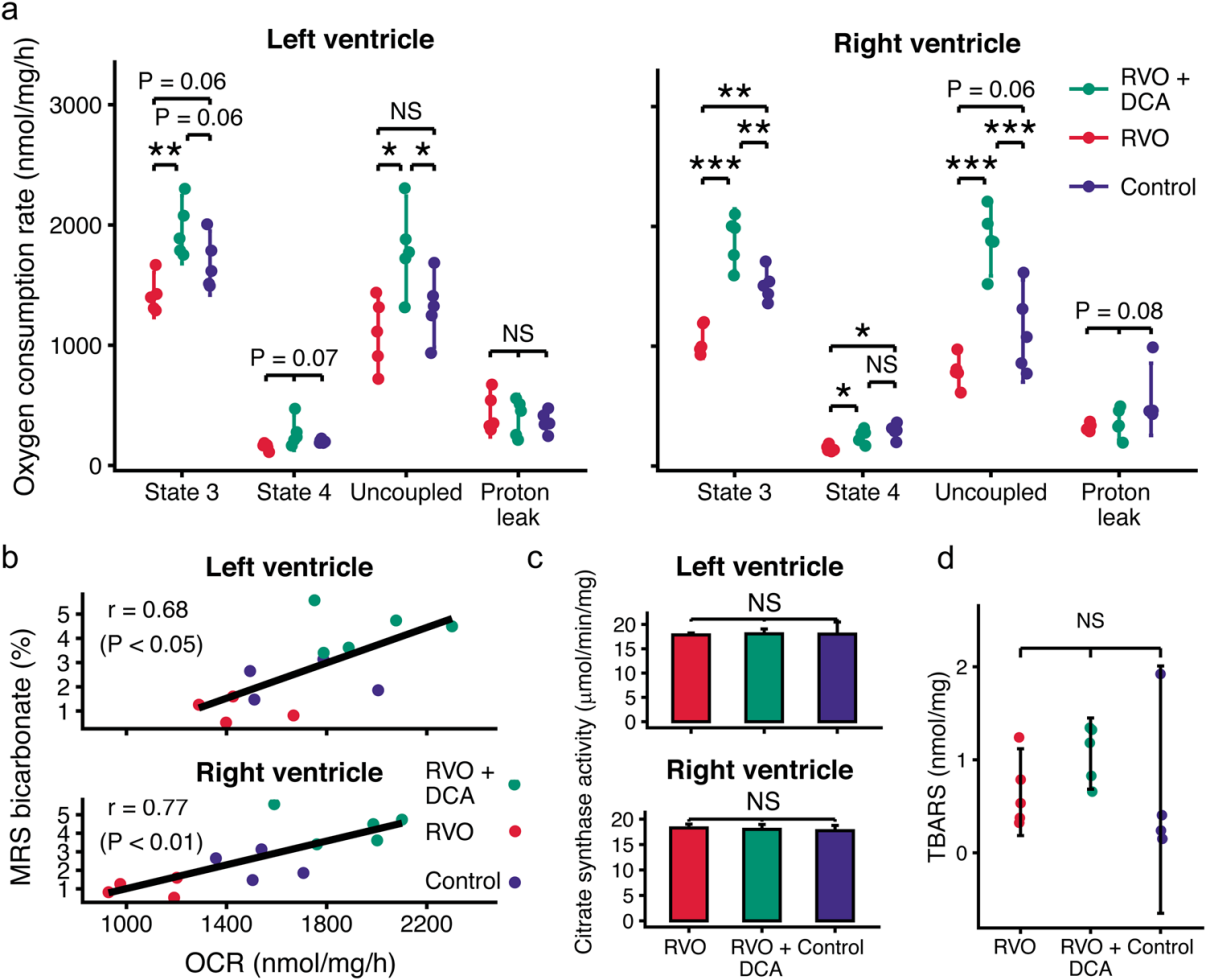
Dichloroacetate (DCA) increases mitochondrial respiratory function after right ventricular volume overload (RVO) in pigs. (**a**) The respiration rates of isolated cardiac mitochondria in a closed-chamber system with pyruvate and malate as substrates after 19 weeks of overload. (**b**) The maximal *in-vitro* mitochondrial state 3 oxygen consumption rates (OCR) correlates with bicarbonate production as determined with hyperpolarized [1-^13^C]pyruvate magnetic resonance spectroscopy (MRS) under dobutamine-induced stress. (**c**) Citrate synthase activity in the isolated mitochondria. (**d**) Thiobarbituric acid reactive substances (TBARS), an end-product of oxidative stress, in the overloaded right ventricle. Error bars indicate 95% confidence intervals. NS: P > 0.1, * P < 0.05, ** P < 0.01. *** P < 0.001, ANOVA with Benjamini-Hochberg correction. Correlation was assessed with Pearson’s method.

As presented in Fig. 4a, the state 3 respiration rate of right ventricular mitochondria from non-treated RVO animals (1058 ± 128 nmol/mg/h) was significantly lower than in the control group (1509 ± 131 nmol/mg/h, P = 0.0012). In the DCA treated group, mitochondrial respiration rate was increased (1888 ± 208 nmol/mg/h, P = 0.0028 vs controls). A similar pattern was observed in mitochondria from the left ventricle, where DCA increased mitochondrial function compared with the non-treated animals (P = 0.0588 vs controls, P = 0.0032 vs non-treated). The *in-vitro* mitochondrial respiration rates showed a strong positive correlation with the *in-vivo* hyperpolarized MRS assessments of PDH flux (Fig. 4b). During stress, the correlations were *r* = 0.77 (P = 0.0022) for the right ventricle and *r* = 0.68 (P = 0.0113) for the left ventricle. At rest, the correlations were *r* = 0.72 (P = 0.0037) for the right ventricle and *r* = 0.67 (P = 0.0087) for the left ventricle (correlations at rest are not shown in Fig. 4b). There were no differences in citrate synthase activity (Fig. 4c). Further, the amount of thiobarbituric acid reactive substances (TBARS), a marker of oxidative stress, was similar in right ventricular tissue across groups (Fig. 4d).

### Expression of metabolic regulators

Gene expressions in right ventricular tissue are presented in Table 2. The expression of the glucose transporters 1 and 4 (GLUT1 + 4) was similar between groups, as was the expression of the monocarboxylate transporter 1 (MCT1) and ANP. Expression of PDK1 and 4 was not significantly altered between groups. Likewise, we observed no differences in protein levels of PDK4 (controls: 0.615 ± 0.11 ng/mg, RVO: 0.575 ± 0.04 ng/mg, RVO + DCA: 0.582 ± 0.2 ng/mg; P = 0.9). Expression of the peroxisome proliferator-activated receptor gamma co-activator 1 alpha (PGC1α), a regulator of mitochondrial biogenesis, the peroxisome proliferator-activated receptor alpha (PPARα) and the carnitine palmitoyl transferase I (CPT1β), both mediators of fatty acid metabolism, was similar between groups. However, expression of the long chain acyl-CoA dehydrogenase (LCAD), an important enzyme in beta oxidation, was lower in the RVO groups (P = 0.011 vs controls).

**Table 2:** Expression of key carbohydrate metabolism genes after 19 weeks of right ventricular volume overload (RVO) with or without dichloroacetate (DCA) treatment relative to controls.

	Expression fold-change	
	RVO (n = 5)	RVO + DCA (n = 4)
Gene		
GLUT1	0.84	0.86
GLUT4	0.76	1.0
MCT1	1.03	0.88
PGC1α	1.18	1.55
PDK1	1.03	1.02
PDK4	1.79	1.74
ANP	1.1	1.02
CPT1β	1.04	1.02
PPARα	0.92	0.79
LCAD	0.8*	0.8*

GLUT = glucose transporter, MCT = monocarboxylate transporter PGC = peroxisome proliferator-activated receptor gamma coactivator, PDK = pyruvate dehydrogenase kinase, ANP = atrial natriuretic peptide, PPARα = peroxisome proliferator-activated receptor alpha, CPT1β = carnitine palmitoyl transferase I, LCAD = long chain acyl-CoA dehydrogenase. Asterisks indicate significance vs the control group in ANOVA with Benjamini-Hochberg correction.

## Discussion

We report that PDK inhibition prevents myocardial hypertrophy and improves the biventricular contractile reserve in a pig model of early right heart failure from volume overload. Mechanistically, DCA increased the PDH flux reserve and improved mitochondrial function without significantly altering the expression of key metabolic transporters and regulators.

The failing heart switches from fatty acid utilisation to glycolysis^1,2^. While increased glucose oxidation has been observed^14^, most data suggest that pyruvate oxidation decreases, leading to uncoupling of glycolysis and mitochondrial metabolism^2–4,15^. Increased pyruvate anaplerosis and ketone utilisation has been shown to accompany this^2,16^. To accommodate this complexity, the functional concepts of metabolic reserve and flexibility were introduced as hallmarks of myocardial metabolism^1,2,11^. In accordance, we investigated heart failure metabolism *in-vivo* as well as *ex-vivo*. To assess the interplay between function and metabolism, we performed simultaneous measurement of contraction, perfusion and metabolism at rest and under dobutamine stress. We found decreased LCAD expression in the overloaded hearts, suggesting a beginning switch from fatty acid utilisation. Using MRS with hyperpolarized pyruvate, we observed deceased PDH flux in the overloaded hearts. As perfusion and expression of the MCT1 pyruvate transporter did not change, our observations were a result of changes in intracellular pyruvate metabolism, not cellular pyruvate availability. Further, hypoxia was unlikely to play a role, as myocardial perfusion and expression of GLUT1 and PDK1, both target genes of the hypoxia inducible factor 1, were unaffected. This is in accordance with observations from human non-ischemic heart failure^17^. Thus, we observed uncoupling of glycolysis from mitochondrial metabolism by factors downstream from perfusion, oxygenation and substrate uptake.

Mitochondrial dysfunction is profoundly involved in heart failure^1,2,9^. In response to increased afterload or pacing, PDK4 up-regulation seems to decrease pyruvate oxidation^3,4,7,8^. We found that the up-regulation was insignificant on both gene and protein levels. This could both be due to the early-stage heart failure in our study and a different nature of volume overload. The decreased PDH flux in this study may be secondary to backward failure of the citric acid cycle^15^. Accordingly, we observed decreased *in-vitro* mitochondrial respiration despite similar measures of mitochondrial biogenesis. TBARS levels was similar between groups, suggesting that the mitochondria were not sufficiently dysfunctional to cause oxidative stress. Others have found that oxidative stress is not present before decompensation^6^. Our findings suggest that compensated mitochondrial dysfunction cause backward failure, leading to uncoupling of glycolysis and mitochondrial metabolism early in heart failure.

It is unknown if there is a metabolic cause for the poor contractile reserve in heart failure. In this study, the controls fuelled stress with a non-specific increase in total pyruvate metabolism. The non-treated animals showed a similar but poor metabolic reserve and had no contractile reserve. In the DCA treated animals, the energy demands of stress were countered by increasing the PDH flux, which was sufficient to fuel a contractile reserve. Increased PDH flux equals increased oxidation of pyruvate, the end-product of cytosolic carbohydrate metabolism. Augmentation of carbohydrate oxidation may improve the contractile reserve through several mechanisms (see Fig. 1 for overview). Upstream of the mitochondria, DCA increases cardiac lactate utilisation and increases metabolic flexibility during exercise. This may protect the mitochondria from damage caused by metabolic inflexibility^18^. In accordance, we found that DCA prevented decrease of mitochondrial respiration. Similarly, Piao and colleagues found that DCA restored the respiration of cardiac mitochondria in a rat model of pulmonary hypertension^8^. By recoupling glycolysis to mitochondrial metabolism, DCA decreases inefficient pyruvate anaplerosis^16^. Downstream of the PDH, DCA may cause stockpiling of acetylcarnitine, which buffers acetyl-CoA and improves the mitochondrial inertia^19,20^. DCA improves exercise capacity in skeletal muscle, supposedly by a similar mechanism^21^. Through these mechanisms, DCA restores the carbohydrate metabolic reserve, which in turn improves the contractile reserve.

Cardiac metabolism and hypertrophy are intimately linked^11,22^. Several studies have demonstrated the antihypertrophic properties of DCA in rodents^6–8^. A human study have shown antiproliferative properties of DCA in the vasculature^23^. In the light of these and our observations, PDK inhibition seems a promising antihypertrophic strategy. Mechanistically, prolonged high rates of glycolysis^18^ and poor mitochondrial health^24^ are involved in pathological remodelling. DCA may work through those or by remodelling of epigenetics^25^, protecting from oxidative stress^6^, decreasing anaplerosis^16^ or improving metabolic flexibility^7^. Further studies are needed to elucidate the precise molecular links and safety of metabolic antihypertrophic therapy.

Isolated right ventricular volume overload is uncommon; however, we chose this model for several reasons. The patients are often young and conventional therapy is ineffective^26^. In the light of increasing prevalence, the mechanisms of right ventricular dysfunction remain understudied^27^. The pulmonary insufficiency of this model causes early stage hyperdynamic heart failure, which is well-characterised using MRI and invasive conductance measurements^28,29^. We observed that ANP was slightly increased in plasma, while its expression was unaltered in right ventricular tissue. This underlies that this model depicts early failure. Similarly, it is in line with the notion that traditional biomarkers of heart failure may be less upregulated in right-sided congenital heart disease^26^. It should be noted that the expression analysis was performed on ventricular tissue, and thus the ANP in plasma may be of atrial origin. The model allowed examination of heart failure metabolism in large animals. The right ventricle may be more susceptible to the metabolic changes in heart failure, as is the case in acute overload^30^, and translation to left-sided overload should be done with care. However, many of our observations were made in both ventricles, and in right ventricular pressure overload, the left ventricular myocardium is affected as well^31^. This supports the idea that no cardiac disease is truly univentricular^32^.

Since hyperpolarized pyruvate was used to visualise human heart metabolism^33^, the potential of the metabolic MRI technique has been under investigation. We observed a solid correlation between mitochondrial respirometry, the gold standard measure of mitochondrial function, and PDH flux measured with hyperpolarized pyruvate MRS. This indicates that the technique can detect compensated mitochondrial dysfunction early in heart failure. Further, we introduce stress metabolic MRI as a potential diagnostic tool of decreased carbohydrate metabolic reserve. We found hyperpolarized pyruvate MRS able to detect decreased PDH flux earlier in heart failure than in the study by Schroeder and colleagues^4^, who used a pig model of right ventricular pacing. This may be due to differences in the MR acquisition, as we used spectroscopy while they performed imaging. However, the technique is constantly improving, and future imaging studies may be able to detect decreased bicarbonate early as in the present study. Our observations enlarge the promise of metabolic MRI to allow clinical examinations of the bioenergetic status of the heart, opening new possibilities for personalised treatment of metabolic impairment in heart failure.

Targeting metabolism may be the future of heart failure therapy^2,9^. We show that PDK inhibition may be a strategy for prevention of hypertrophy and mitochondrial dysfunction. While previous studies mostly concerned end-stage disease^6,8^, we observed that the therapeutic effect is prevalent early. As mitochondrial dysfunction is a hallmark in decompensation^9,24^, treatments that prevent mitochondrial dysfunction are attractive. Further, PDK inhibition may improve exercise capacity by improving the contractile reserve. Previously, DCA administration has been attempted in acutely decompensated patients with conflicting results^34,35^. Considering its therapeutic effects - reduced hypertrophy, increased metabolic reserves and better contractile reserves - DCA seems more relevant in chronic heart failure. After reluctance to long-term DCA use in humans, a recent trial showed that DCA attenuate vascular remodelling in pulmonary arterial hypertension^23^.

In summary, we show that DCA reduces hypertrophy, protects the mitochondria and improves the contractile reserve by increasing the carbohydrate metabolic reserve. In light of the reformed interest in PDK inhibition, this approach seems worth investigating in chronic heart failure, which, to our knowledge, has never been done.

## Methods

### Animal model

We established pulmonary regurgitation in Danish landrace pigs (n = 10, 15.98 ± 1.63 kg, female) by external suturing of the anterior pulmonary valve leaflet. Over time, the volume overload causes ventricular dilation and heart failure^28,29^. In week 12, the pigs were randomised to DCA (n = 5, 50 mg/kg/day) or no treatment (n = 5). The DCA was administered orally in capsules twice a day for seven weeks. DCA inhibits the PDKs. In week 19, we introduced weight matched controls and performed imaging and tissue sampling. An experimental timeline is provided online (Fig. S1). Propofol (4 mg/kg/h IV) and fentanyl (0.035 mg/kg/h IV) were used for anaesthesia. Depth of anaesthesia was monitored using invasive blood pressure, heart rate measurements and corneal and ciliary reflexes. Post-operative analgesia was a Bupivacaine block, paracetamol (250 mg × 2 orally for three days) and Meloxicam (0.4 mg/kg × 1 IM for three days). Sacrifice was carried out with exsanguination under profound anaesthesia. The investigation was approved by the Danish Animal Inspectorate (2016-15-0201-01061) and conforms to current European legislation (Directive 2010/63/EU).

### Magnetic resonance imaging and spectroscopy

The pigs were fed and received an oral glucose bolus (2 g/kg) 3.5 hours and 45 minutes before MRS, respectively. MRI and hyperpolarized [1-^13^C]pyruvate MRS were carried out at rest and under dobutamine-stress (10 µg/kg/min IV), which is used for mimicking exercise in the clinic^36^. All examinations were carried out on a commercial 3 T MRI system (GE Healthcare). The [1-^13^C]pyruvate was hyperpolarized in a SPINLab polarizer (GE Healthcare)^37^. At the start of pyruvate injection, 128 spectra were acquired. After post-processing^38^, pyruvate to metabolite conversion was quantified using the area under the curve approach^39^. Myocardial pH was estimated from the bicarbonate and lactate signals^40^. Conventional MRI was analysed with segmentation and volume measures were normalised to body weight^41^. The MRS stress data of one RVO animal and the stress data of one control animal were excluded due to equipment failure.

### Blood samples

Glucose and lactate were determined using point-of-care equipment (ABL90 FLEX PLUS, Radiometer Medical). Free fatty acids, insulin and ANP were measured with colorimetric assays (Sigma Aldrich).

### Mitochondrial respirometry

After sacrifice, the respiration of isolated cardiac mitochondria was assessed in a closed chamber system (Unisense). Myocardial samples were homogenised in isolation medium (250 mM sucrose, 10 mM HEPES, 1 mg/ml BSA_fatty acid free_, pH 7.4, 300 mOsm/kg H_2_O). Using centrifugation, debris was removed (800 g for 10 min) and the mitochondria were isolated (14500 g for 5 min). The dead mitochondria were rinsed away, and the intact mitochondria were recentrifuged and resuspended in preservation medium (250 mM Sucrose, 5 mM HEPES, pH 7.4, 300 mOsm/kg H_2_O). Before respirometry, mitochondria were suspended in MIR05 respiration media (given in mM: 110 sucrose, 60 K-lactobionate, 0.5 EGTA, 0.1% BSA, 3 MgCl_2_, 20 taurine, 10 KH_2_PO_4_ and 20 HEPES; pH 7.1). All values were normalised to protein content. Respiration states were determined with addition of the following: state 4 with pyruvate (10 mM) and malate (2 mM), state 3 with ADP (5 mM) in the presence of pyruvate and malate, leak respiration with oligomycin A (2 µg/mL), uncoupled respiration with carbonyl cyanide-p-trifluoromethoxyphenylhydrazone (1.5–2 µM) and non-mitochondrial respiration with rotenone (0.5 µM) and antimycin A (2.5 µM).

### Enzyme activity

LDH activity, PDH activity, citrate synthase activity and TBARS were determined in myocardial biopsies (isolated mitochondria for citrate synthase) with colorimetric assays (Sigma Aldrich) and normalised to protein content.

### Right ventricular gene and protein expression

RNA was extracted using TRIzol Reagent, reverse transcription was performed using the Applied Biosystems High-Capacity cDNA Reverse Transcription Kit and quantitative PCR was performed on a 7500 Fast Real-Time PCR System under standard conditions using TaqMan Fast Universal PCR Master Mix (Thermo Fisher Scientific). Each biological sample was run in technical duplicates for each gene. The three most stable reference genes (B2M, ACTB and HPRT1) were chosen from 5 candidates. Fatty acid metabolism genes were analysed using SYBR green reagents and 18S as the reference. Primers are described in Table S3 of the Supplementary Information.

Myocardial PDK4 protein content was assessed using a colorimetric assay (LSBio) and normalized to protein content.

### Statistics

All data are presented as means (SD) if not otherwise specified. Significance was considered at P < 0.05. Groups were compared using analyses of variance (ANOVA) or Kruskal-Wallis tests as appropriate. In case of significance, two-sided t-tests with Benjamini-Hochberg correction for multiple comparisons were performed. Interactions between group and rest or stress state were assessed using repeated measures ANOVA. Analyses of magnetic resonance data, tissue and plasma were carried out blinded. All analyses were performed using the R statistical environment. Data are available upon request to the authors.

## Supplementary information

Supplementary information.
