# Supplementary material for: Increasing carbohydrate oxidation improves contractile reserves and prevents hypertrophy in porcine right heart failure
Source: Sci Rep. 2020 May 18;10:8158. doi: 10.1038/s41598-020-65098-7 (PMC7235019; doi:10.1038/s41598-020-65098-7)
Supplement: Supplementary file 1 — Supplementary information. [file 41598_2020_65098_MOESM1_ESM.pdf]

Supplementary Information:

Title: Increasing carbohydrate oxidation improves contractile reserves and prevents hypertrophy in porcine right heart failure

Short title: Increasing the metabolic reserve in heart failure

Authors: Nikolaj Bøgh[1,2], Esben S. S. Hansen[2], Camilla Omann[1], Jakob Lindhardt[2], Per M. Nielsen[2†], Robert S. Stephenson[3,4], Christoffer Laustsen[2], Vibeke E. Hjortdal[1], Peter Agger[3]

1) The Department of Cardiothoracic and Vascular Surgery, Aarhus University Hospital, Palle Juul-Jensens Boulevard 99, 8200 Aarhus N, Denmark.
2) The MR Research Centre, Department of Clinical Medicine, Aarhus University, Palle Juul-Jensens Boulevard 99, 8200 Aarhus N, Denmark.
3) Comparative Medicine Lab, Department of Clinical Medicine, Aarhus University, Palle Juul-Jensens Boulevard 99, 8200 Aarhus N, Denmark.
4) Institute of Clinical Sciences, College of Medical and Dental Science, The University of Birmingham, United Kingdom.
*) Please address correspondence to Nikolaj Bøgh at email nikolaj.boegh@clin.au.dk, phone 0045 22552808 or the address above.

	RVO (n = 5)	RVO + DCA (n = 5)	Control (n = 5)
Weight (kg)	57.1 (4.1)	57.5 (2.8)	53.3 (1.6)
Follow-up time (days)	131 (4.5)	137 (10.5)	-
Regurgitation (%)	22.4* (10)	20.7* (6.5)	0.2 (0.3)
LV EDV (mL)	95.6 (8.3)	93.1 (10.6)	108.3 (20.9)
RV EDV (mL)	153.9* (36.6)	124.2* (19.7)	77.3 (15.2)
LV ESV (mL)	20.9* (6.3)	26* (3.3)	43.4 (11.8)
RV ESV (mL)	45.3 (17.9)	45.9 (12)	32.3 (8.9)
LV SV (mL)	74.8 (5.7)	67.1 (8.1)	65 (9.7)
RV SV (mL)	108.6*† (18.8)	78.4* (12.7)	45 (7.4)
LA size (mL)	57.9 (14.3)	46 (8.5)	61.2 (5.8)
RA size (mL)	64.7 (8.2)	60.9 (22.1)	58.7 (8.9)
Ecc. index systole	0.96 (0)	1.04 (0.1)	0.99 (0.1)
Ecc. Index diastole	1.29* (0.1)	1.29* (0.1)	1.11 (0)
QRS duration (ms)	86* (0.6)	85* (12)	63 (0.5)

Table S1: Characteristics in week 19 of control pigs and pigs subjected to right ventricular volume overload (RVO) without and with dichloroacetate (DCA) treatment. Asterisks indicate significance vs control group, daggers indicate RVO vs RVO + DCA significance tested using ANOVA with Benjamini-Hochberg correction. LV = left ventricle, RV = right ventricle, EDV = end-diastolic volume, ESV = end-systolic volume, SV = stroke volume, LA = left atrial, RA = right atrial, ecc. index = eccentricity index (anterior-to-posterior distance relative to septum-to-free wall distance).

	Rest			Stress		
	RVO (n = 5)	RVO + DCA (n = 5)	Control (n = 4)	RVO (n = 5)	RVO + DCA (n = 5)	Control (n = 3)
Left ventricle						
Circumferential strain	-29.4* (3.8)	-26.3* (3.5)	-19.5 (2.4)	-24.1* (3.7)	-24.8* (3.0)	-17.3 (1.5)
Circumferential SR, peak systole (s^{-1})	-87.9 (13.7)	-88.6 (17.4)	-77 (12.4)	-129.4† (13.6)	-173.8* (32.5)	-120.5 (4.5)
Circumferential SR, early diastole (s^{-1})	126.2 (26.2)	125 (37)	80.4 (21.2)	170.7 (24)	180.6 (24.5)	165.7 (17.7)
Radial strain	62.7* (6.8)	55.7* (11.8)	35.8 (1.9)	69.8 (9.6)	62.1 (23.2)	37.8 (8.4)
Radial SR, peak systole (s^{-1})	179.5 (21.6)	174.6 (46.1)	132.8 (5.9)	337.4 (40.9)	407.2* (58.5)	253.4 (45.1)
Radial SR, early diastole (s^{-1})	-278.1* (4)	-267.2* (90.7)	-154.7 (25.6)	-388.6 (68.2)	-427 (162.8)	-310.2 (68.9)
Right ventricle						
Circumferential strain	-16.7* (2.6)	-15* (3.7)	-9 (3.4)	-14.7 (4.8)	-15.6 (2.7)	-11.4 (1.5)
Circumferential SR, peak systole (s^{-1})	-48.9 (2.6)	-46 (13.2)	-31.8 (10.7)	-72.8 (24.5)	-93.6 (23.5)	-81.3 (12.9)
Circumferential SR, early diastole (s^{-1})	66.1 (12.2)	72.8 (30.8)	42.7 (11.5)	81.8 (17.4)	104.3 (20.3)	110.6 (27.1)

Table S2: MRI feature-tracking strain measurements from pigs subjected to right ventricular volume overload (RVO) receiving no treatment or dichloroacetate (DCA). Stress was dobutamine-induced (10 mcg/kg/min). One control animal was excluded due to insufficient data quality. Asterisks indicate significance vs control group, daggers indicate RVO vs RVO + DCA significance tested using Kruskal-Wallis with Benjamini-Hochberg correction.

SR = strain rate.

Gene	TaqMan kit/primers
B2M	Ss03391156_m1
ACTB	Ss03376081_u1
HPRT1	Ss03388274_m1
GLUT1	Ss03374747_s1
GLUT4	Ss03373325_g1
MCT1	Ss03374095_m1
PGC1A	Ss03393114_u1
PDK1	Ss04246164_m1
PDK4	Ss03822089_s1
ANP	Ss03394501_g1
CPT1β	CTGGCCTTCCAATTCACAGT<br>AGTAGGAGGAACCCGCTGTT
PPARα	CCATGCTGTCCTCTGTGATG<br>CCAAATGATAGCAGCCACAA
LCAD	TTGGAGGGGACTTGTACTCG<br>TCCAGGCTCTGTCATTGCTA
18S	ACGCTGAGCCAGTCAGTGTA<br>GTGGAGCGATTTGTCTGGTT

Table S3: Primers used for RT-qPCR.

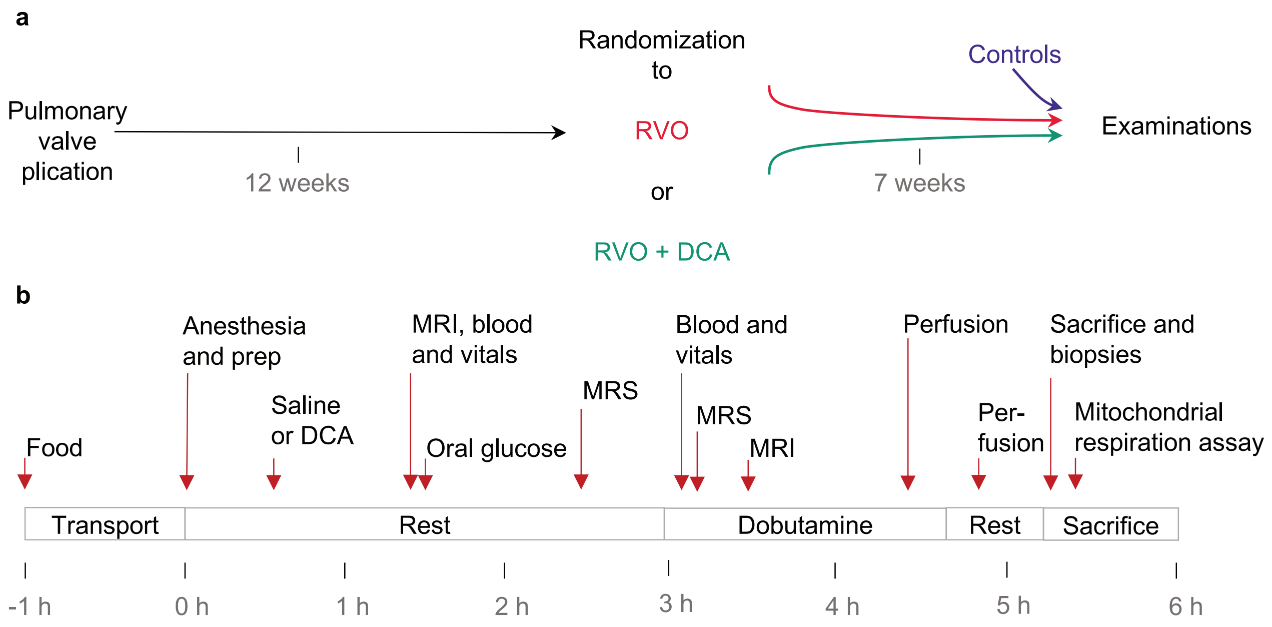

Figure S1: Examination of myocardial metabolism and cardiac function after chronic volume overload of the right ventricle (RVO). **a**: Overview of the study. After 12 weeks of overload, we randomised the animals to dichloroacetate (DCA) or nothing. After a total of 19 weeks, we included weight-matched controls and examined all groups. **b**: Overview of the examinations, that included magnetic resonance imaging (MRI) and hyperpolarized [1-^{13}C]pyruvate spectroscopy (MRS) under rest and dobutamine induced stress (10 mcg/kg/min).
